# Shoulder Instability. Current Concepts and Controversies

**DOI:** 10.2174/1874325001711010810

**Published:** 2017-08-31

**Authors:** Miguel Angel Ruiz Ibán

**Affiliations:** 1 Hospital Universitario Ramon y Cajal Cta Colmenar km9 100, Madrid 28046, Spain

Shoulder instability is a highly prevalent problem that affects mainly young and able individuals. As such, its impact in the quality of life of the general population is significant. Its evaluation and management have traditionally been controversial: the pathoanatomy, the clinical [1] and radiologic [2] evaluation and the choice of an adequate treatment, even before the advent of arthroscopic surgery [3], are heavily disputed topics. Most of the different approaches to the problem, from defining what is the best conservative treatment after a first episode [4] to selecting the most appropriate surgical technique, are disputed between orthopedic surgeons and constantly under review [5]. Notwithstanding that, in the last few years, a lot of very valuable information has been published that is relevant to the topic.

The purpose of the current thematic issue is to focus on both well-stablished knowledge and controversial issues. To do that, there will be a mixture of traditional revision papers that summarize current knowledge and “Controversies” reviews in which the authors will present conflicting literature evidence in a light that is worthwhile for the reader.

The first part includes three reviews that focus in general topics like anatomy [6], evaluation [7] and imaging [8] of the unstable shoulder. All these are well-trodden topics but there are constant innovations in all three fields so a review should be welcomed.

The second part is focused in anterior instability with four reviews of the natural history [9], management alternatives of bone defects [10, 11] and soft tissues [12] in instability and a fifth over the failed shoulder instability patient [13]. Three “controversies” papers focus in specially disputed topics: management of the first shoulder dislocation [14], open or arthroscopic procedures [15] and associated procedures during Bankart repair [16].

The third part of the issue will focus in multidirectional and posterior instability dealing with a general outline of the diagnosis and treatment in four reviews [17-20].

Finally the issue closes with two separate papers focusing in rehabilitation strategies in shoulder instability [21] and anesthetic management [22] of these patients.

The editors hope that this monographic issue will allow the advanced reader in clarifying what does the most current evidence on shoulder instability controversies. It should also be a good reference guide to review the basics of shoulder instability.
